# Supplementary material for: Synthesis, Chiral Resolution and Enantiomers Absolute Configuration of 4-Nitropropranolol and 7-Nitropropranolol
Source: Molecules. 2022 Dec 21;28(1):57. doi: 10.3390/molecules28010057 (PMC9822233; doi:10.3390/molecules28010057)
Supplement: Supplementary file 1 [file molecules-28-00057-s001.zip › molecules-2043157-supplementary.pdf]

Supporting Information

Synthesis, Chiral Resolution and Enantiomers Absolute Configuration of 4-Nitropropranolol and 7-Nitropropranolol

Rosa Sparaco,[1] Antonia Scognamiglio,[1] Angela Corvino,[1] Giuseppe Caliendo,[1] Ferdinando Fiorino,[1] Elisa Magli,[2] Elisa Perissutti,[1] Vincenzo Santagada,[1] Beatrice Severino,[1] Paolo Luciano,[1] Marcello Casertano,[1] Anna Aiello,[1] Gilberto De Nucci,[3,*] Francesco Frecentese[1]

[1] *Department of Pharmacy, University of Naples Federico II, Via D. Montesano 49, Naples, Italy*

[2] *Department of Public Health, University of Naples Federico II, Via D. Montesano 49, Naples, Italy*

[3] *Department of Pharmacology, Faculty of Medical Sciences, State University of Campinas (UNICAMP), São Paulo, Campinas, Brazil*

Content:

Chiral analytical HPLC: stereochemical purity and LC/MS analyses for 4-nitropropranolol and 7-nitropropranolol enantiomers……………… ……..…….. pages S2-S9
Mono and bidimensional NMR spectra of 4-nitropropranolol **2a** and 7-nitropropranolol **2b**……………..…………………………………………...........pages S10-S15
^{1}H-NMR, ^{13}C-NMR and HRMS spectra for bis-MPA derivatives of 4-nitropropranolol and 7-nitropropranolol…………………………..……………………………………… pages S16-S27

Peak 1 - *(+)*-4-Nitropropranolol

Analysis results:

Item	Specification	Result
**Appearance**	Yellow powder	Compliant
**Purity**	LC-MS purity >95%	Compliant
**Enantiomeric purity**	HPLC 99%	Compliant

Stereochemical purity

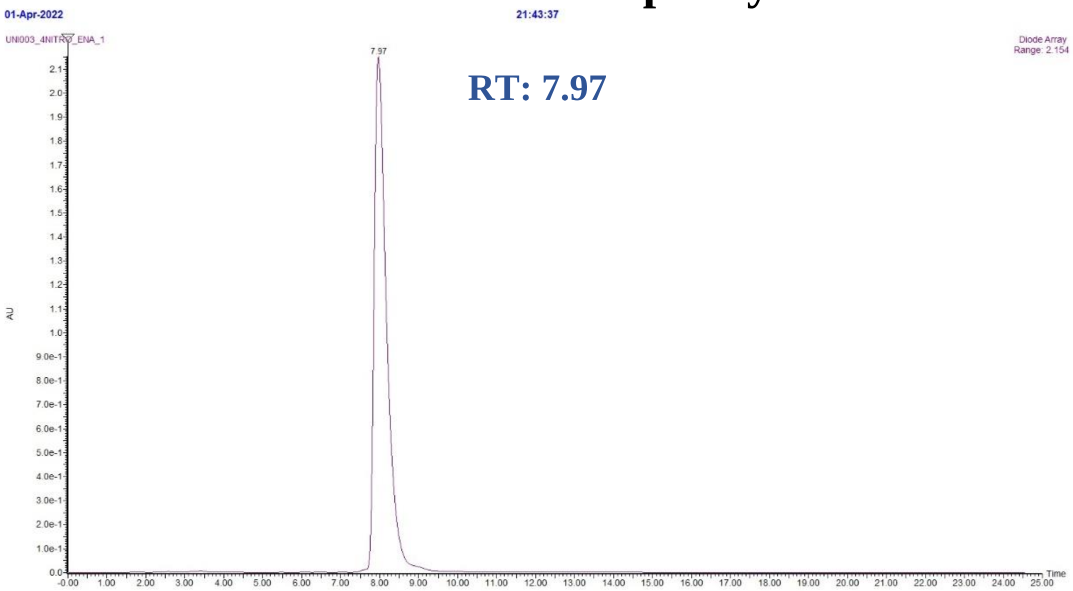

Figure S1 – chiral analytical HPLC: analysis of (+)-4-nitropropranolol

**Sample:**	4-Nitropropranolol – Peak 1	**Flow:**	1 mL/min
**Column:**	5 AMY-Coat -5 µm Phenomenex	**Wavelength:**	254/280 nm
**Dimension:**	150 x 4.6 mm	**Mobile Phase:**	N-Hexane-Isopropanol 86:14 (+0.1% DEA)

LC-MS Analysis - Peak 1 - (+)-4-Nitropropranolol

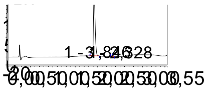

Peak Name	Retention Time min	Area mAU*min	Relative Area %
**1**	1.846	0.046	0,52
**2**	1.923	6.63	98.6
**3**	2.38	0.055	0.82

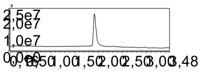

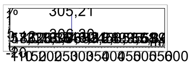

Peak 2 - *(-)*-4-Nitropropranolol

Analysis results:

Item	Specification	Result
**Appearance**	Yellow powder	Compliant
**Purity**	LC-MS purity >95%	Compliant
**Enantiomeric purity**	HPLC 99%	Compliant

Stereochemical purity

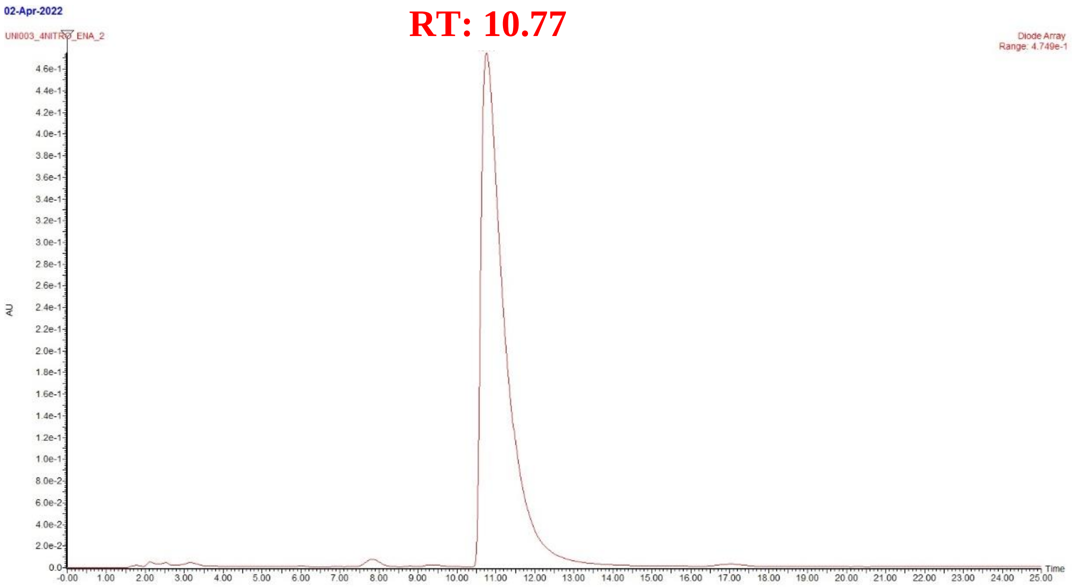

Figure S2 – chiral analytical HPLC: analysis of (*l*)-4-nitropropranolol

**Sample:**	4-Nitropropranolol – Peak 2	**Flow:**	1 mL/min
**Column:**	5 AMY-Coat -5 µm Phenomenex	**Wavelength:**	254/280 nm
**Dimension:**	150 x 4.6 mm	**Mobile Phase:**	N-Hexane-Isopropanol 86:14 (+0.1% DEA)

LC-MS Analysis

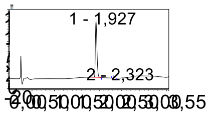

Peak Name	Retention Time min	Area mAU*min	Relative Area %
**1**	1.927	4.36	99.4
**2**	2.323	0.02	0.58

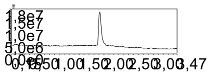

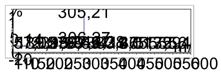

Peak 1 - *(+)*-7-Nitropropranolol

Analysis results:

Item	Specification	Result
**Appearance**	Yellow powder	Compliant
**Purity**	LC-MS purity >95%	Compliant
**Enantiomeric purity**	HPLC 99%	Compliant

Stereochemical purity

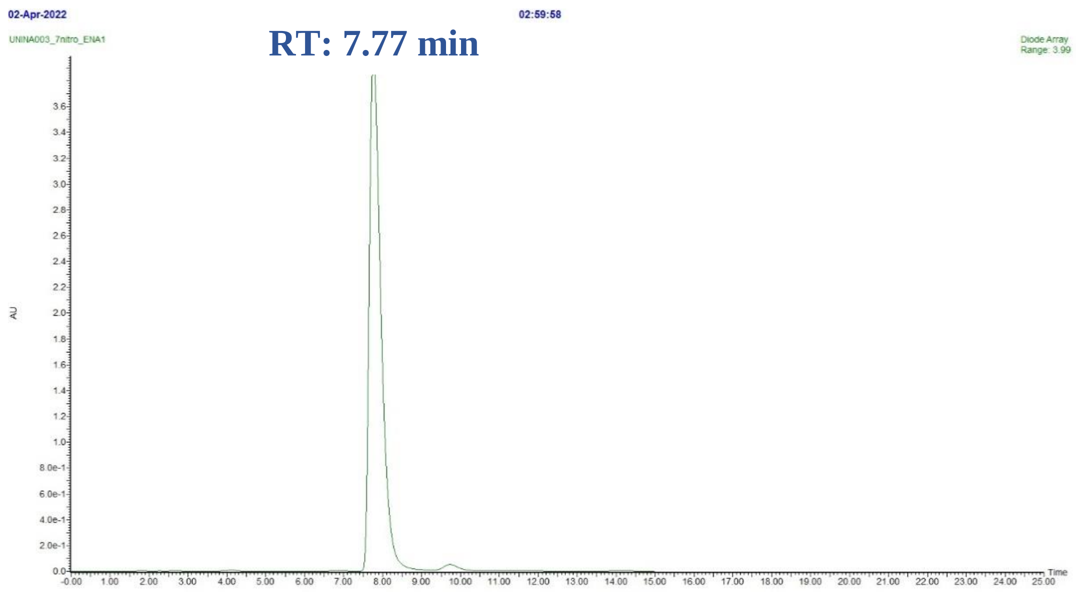

Figure S3 – chiral analytical HPLC: analysis of (d)-7-nitropropranolol

**Sample:**	7-Nitropropranolol – Peak 1	**Flow:**	1 mL/min
**Column:**	5 AMY-Coat -5 µm Phenomenex	**Wavelength:**	254/280 nm
**Dimension:**	150 x 4.6 mm	**Mobile Phase:**	N-Hexane-Isopropanol 86:14 (+0.1% DEA)

LC-MS Analysis

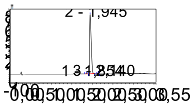

Peak Name	Retention Time<br>min	Area<br>mAU*min	Relative Area<br>%
**1**	1.854	0.077	0.24
**2**	1.945	31.29	99.4
**3**	2.140	0.12	0.39

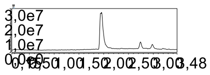

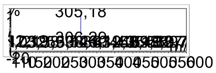

Peak 2 - *(-)*-7-Nitropropranolol

Analysis results:

Item	Specification	Result
**Appearance**	Yellow powder	Compliant
**Purity**	LC-MS purity >95%	Compliant
**Enantiomeric purity**	HPLC 99%	Compliant

Stereochemical purity

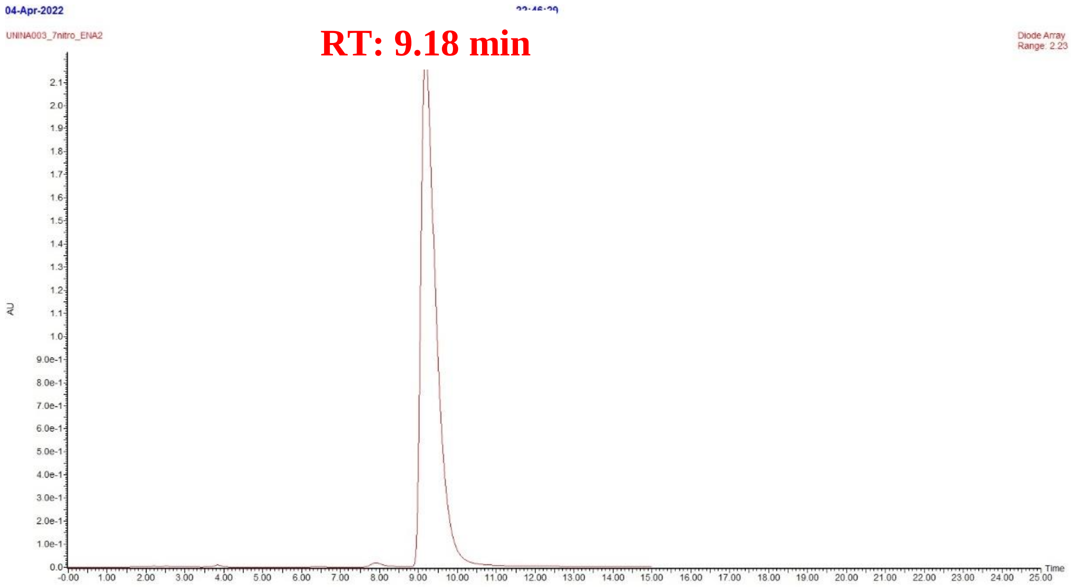

Figure S4 – chiral analytical HPLC: analysis of (l)-7-nitropropranolol

**Sample:**	7-Nitropropranolol – Peak 2	**Flow:**	1 mL/min
**Column:**	5 AMY-Coat -5 µm Phenomenex	**Wavelength:**	254/280 nm
**Dimension:**	150 x 4.6 mm	**Mobile Phase:**	N-Hexane-Isopropanol 86:14 (+0.1% DEA)

LC-MS Analysis

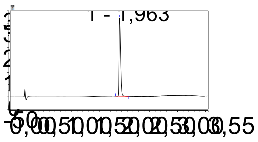

Peak Name	Retention Time min	Area mAU*min	Relative Area %
**1**	1.963	10.13	100

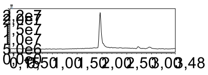

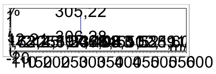

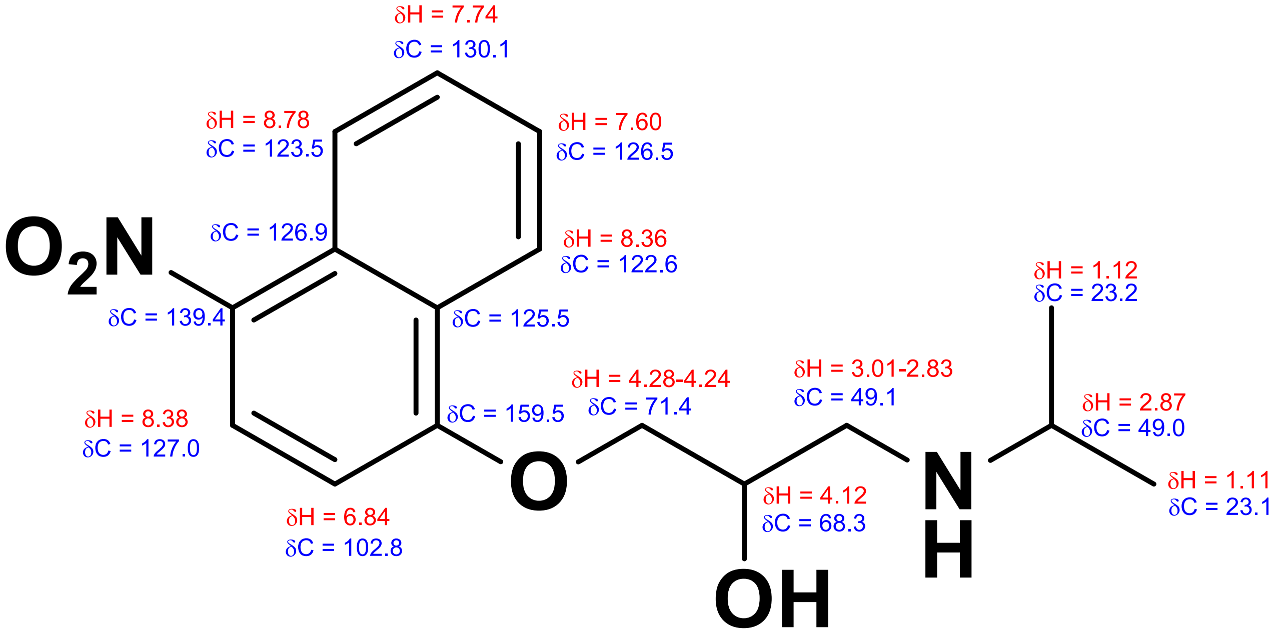

Figure S5 – NMR signals of 4-nitropropranolol **2a**. NMR data obtained from racemic mixture, (+)-enantiomer and (-)-enantiomer are the same.

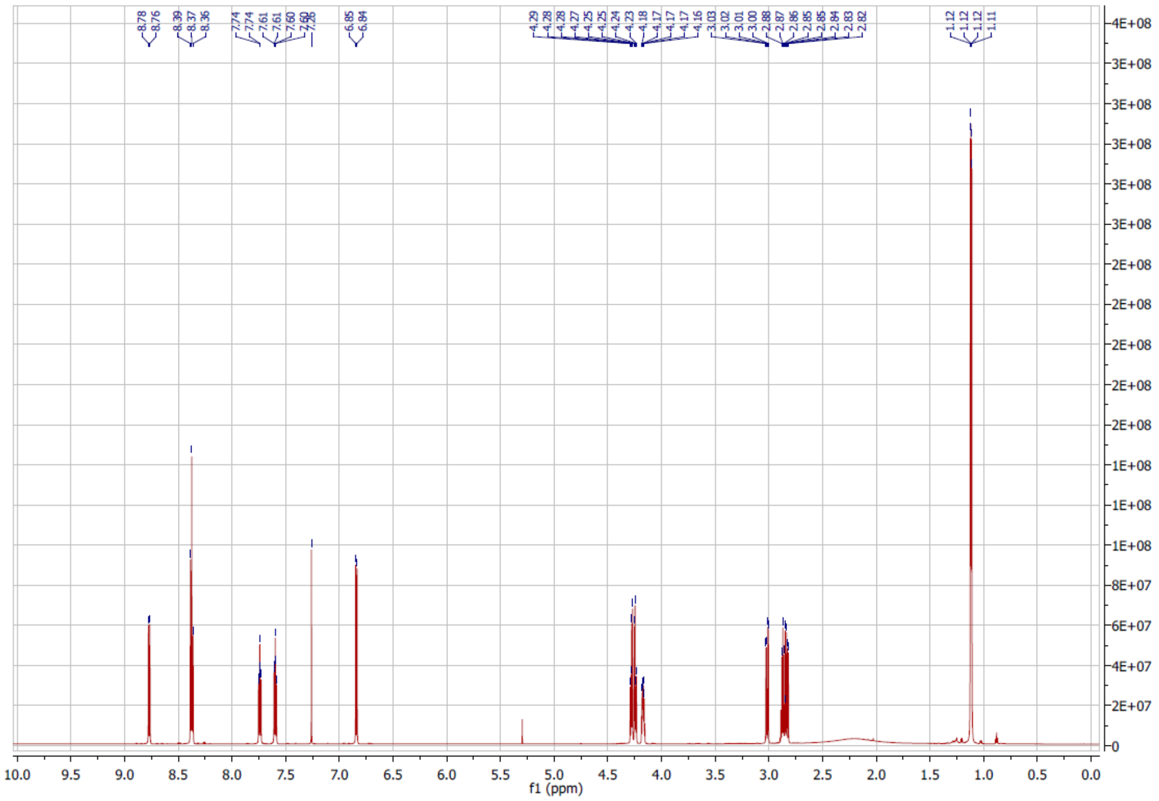

Figure S6 - ^{1}H-NMR 4-Nitropropranolol ($CDCl_3$)

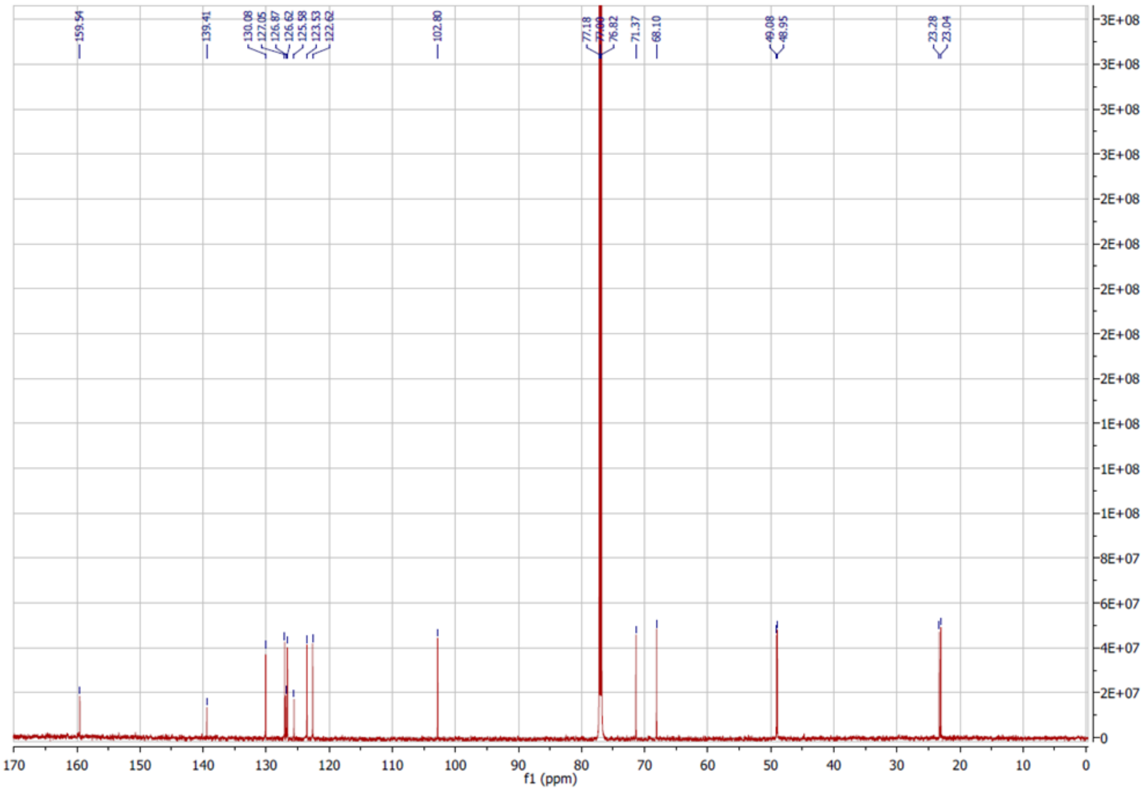

Figure S7 - ^{13}C-NMR 4-Nitropropranolol ($CDCl_3$)

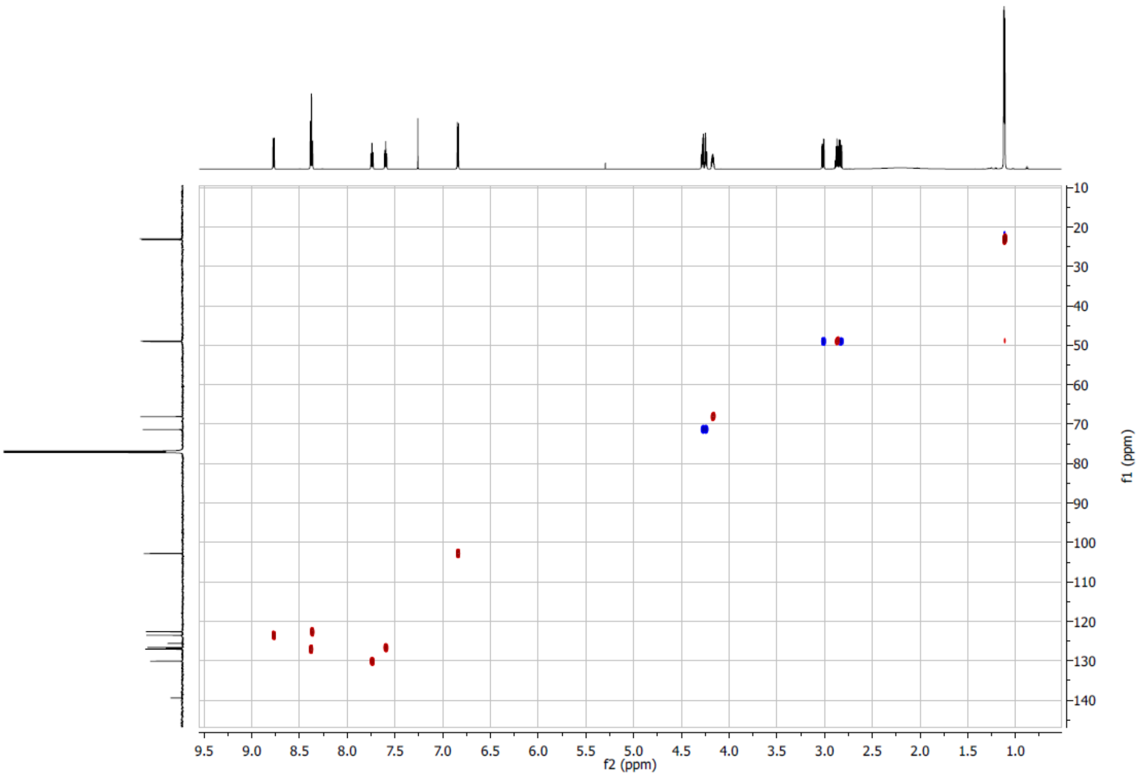

Figure S8 - HSQC 4-Nitropropranolol

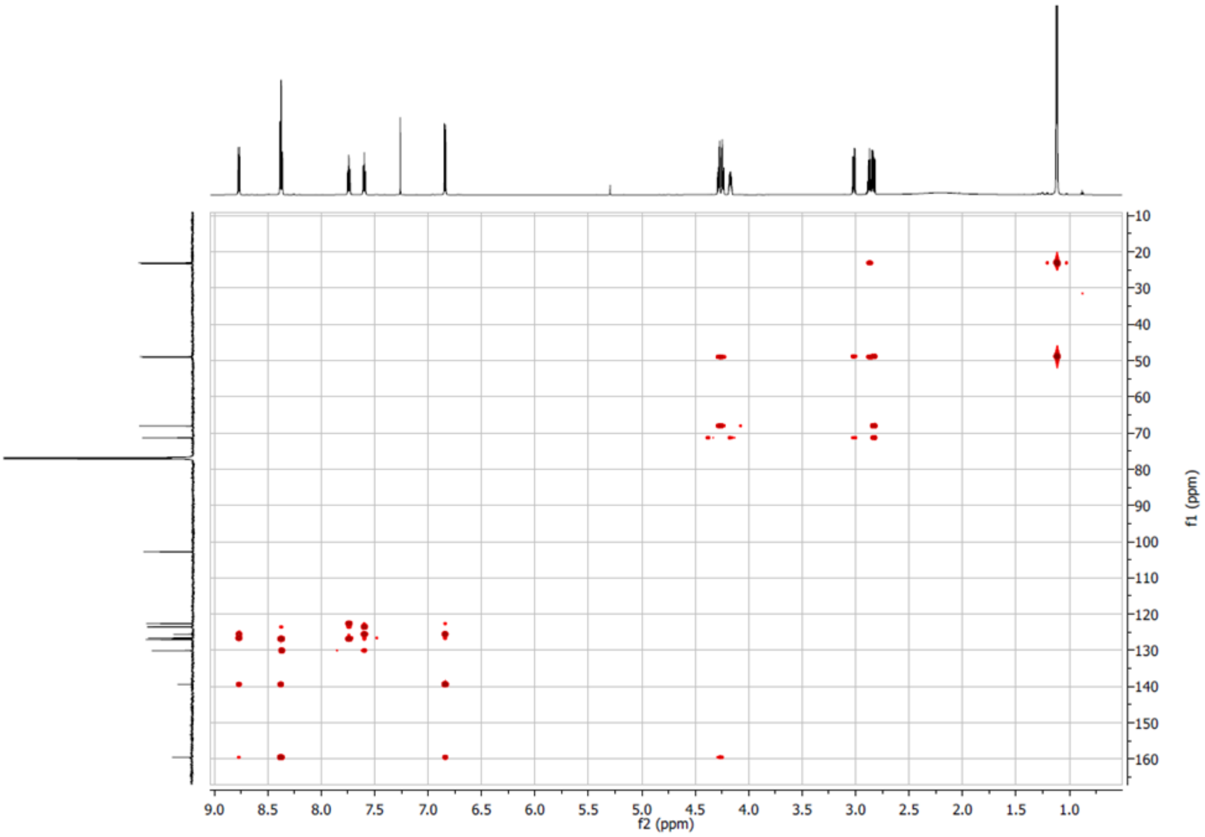

Figure S9 - HMBC 4-Nitropropranolol

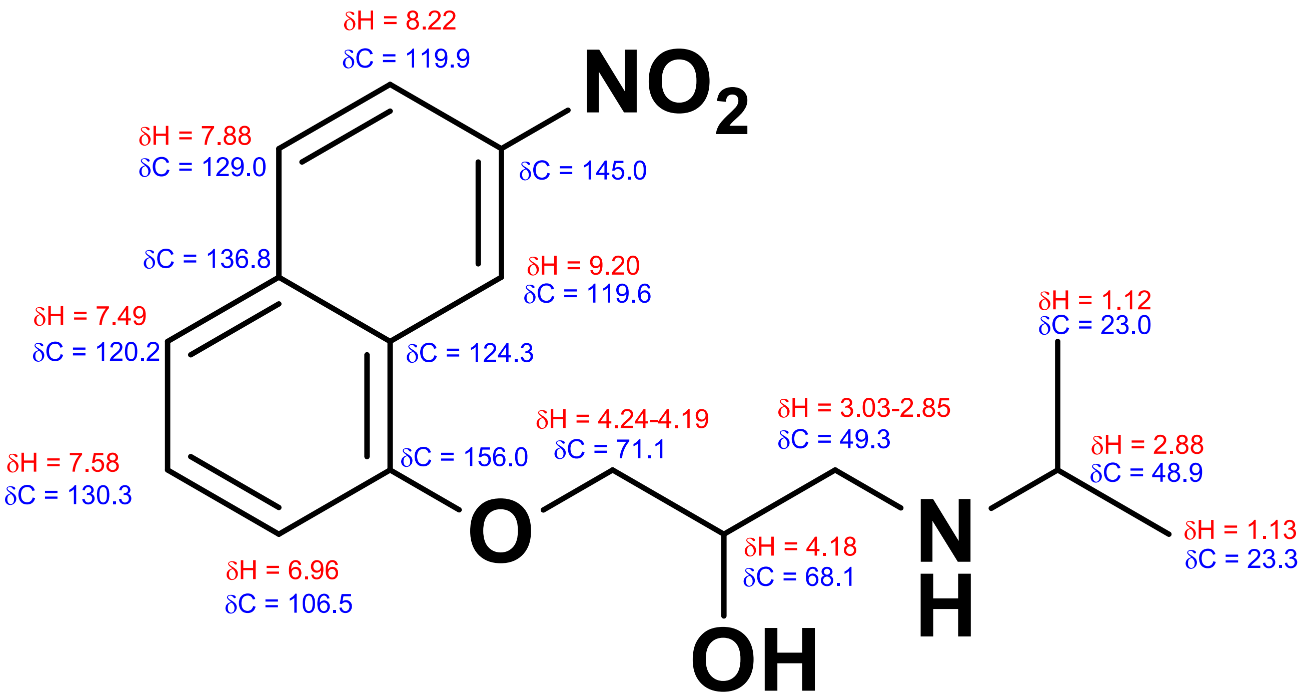

Figure S10 – NMR signals of 7-nitropropranolol **2b**. NMR data obtained from racemic mixture, (+)-enantiomer and (-)-enantiomer are the same.

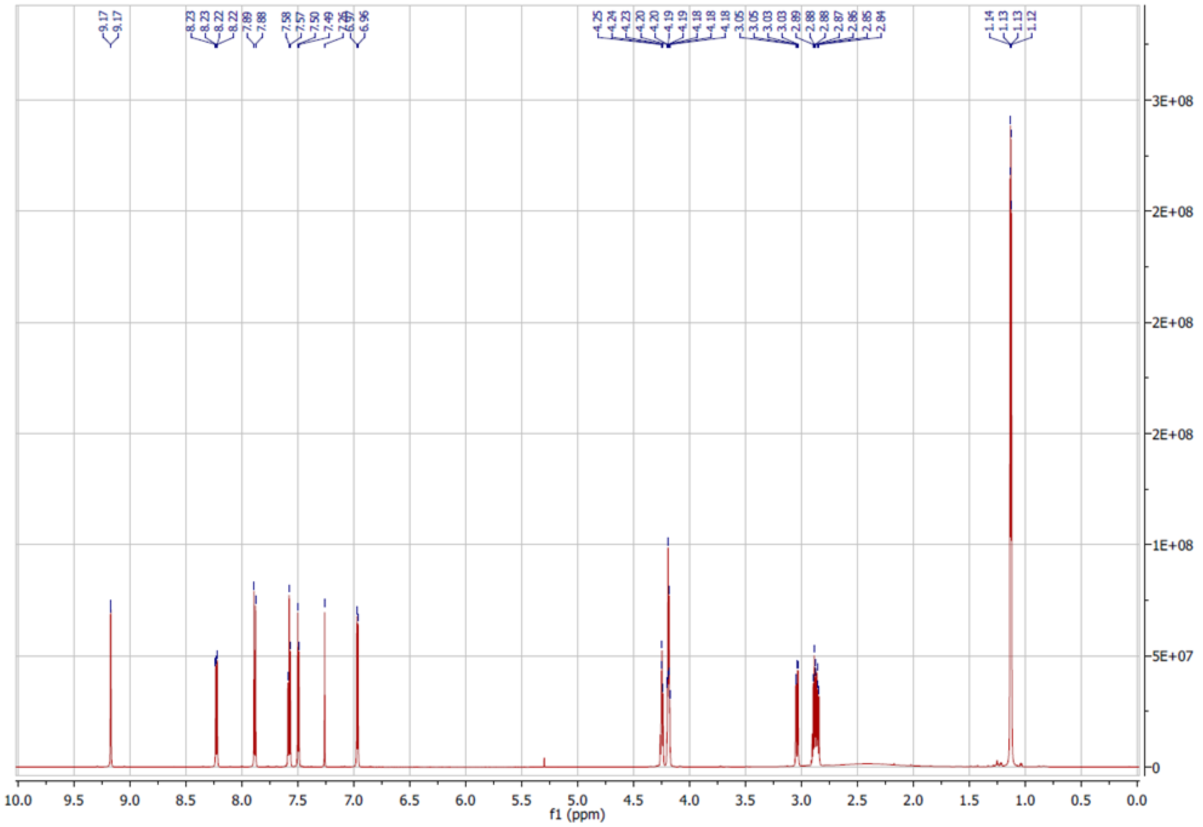

Figure S11 - ^{1}H-NMR 7-Nitropropranolol ($CDCl_3$)

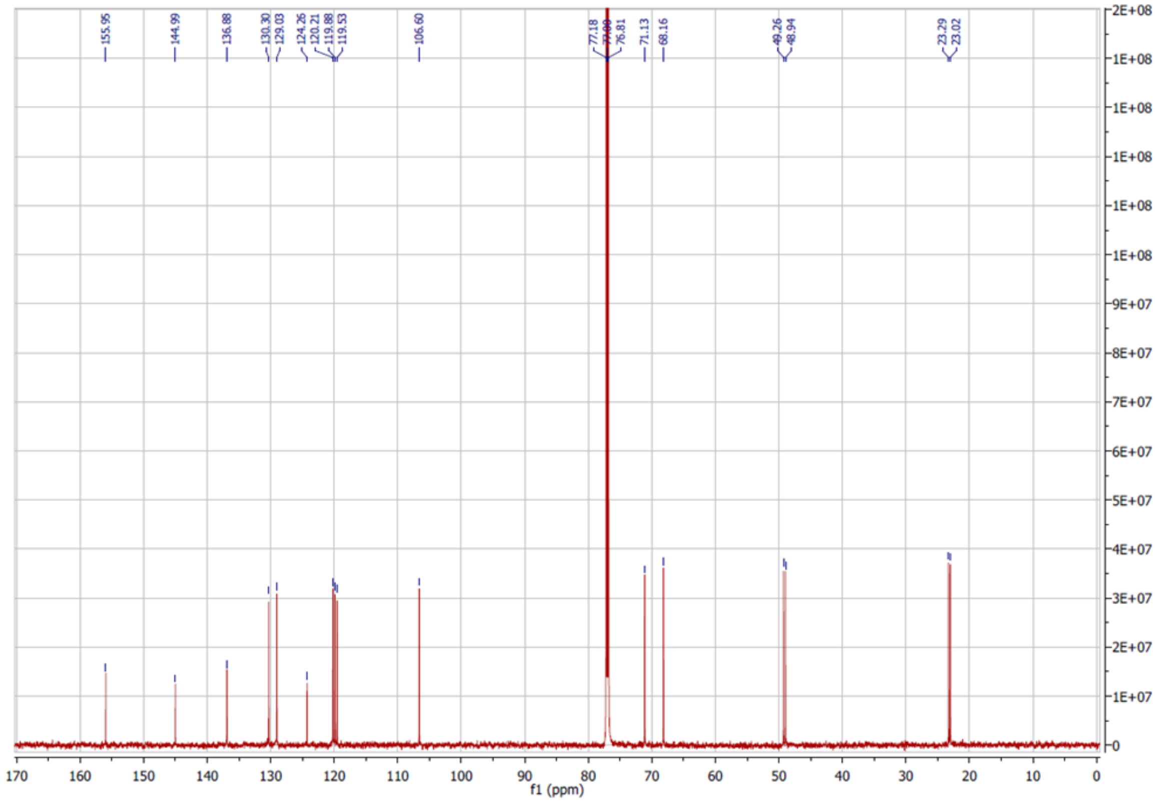

Figure S12 - ^{13}C-NMR 7-Nitropropranolol ($CDCl_3$)

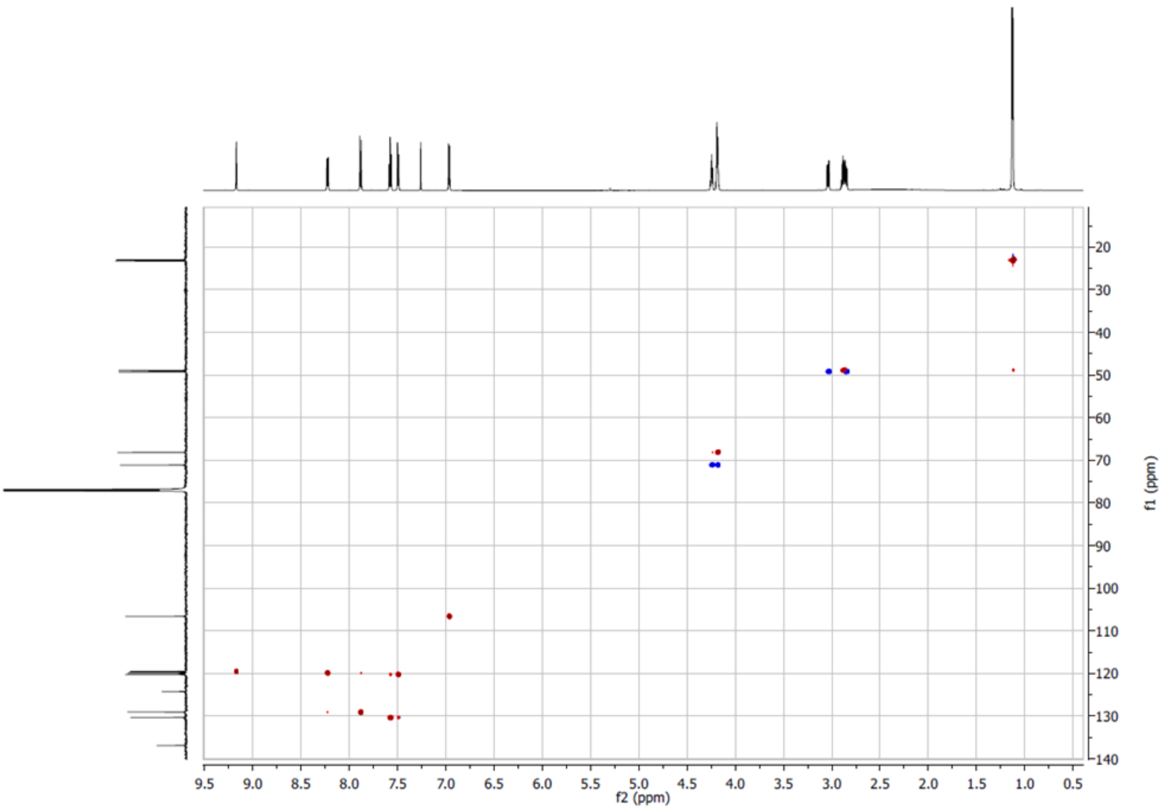

Figure S13 - HSQC 7-Nitropropranolol

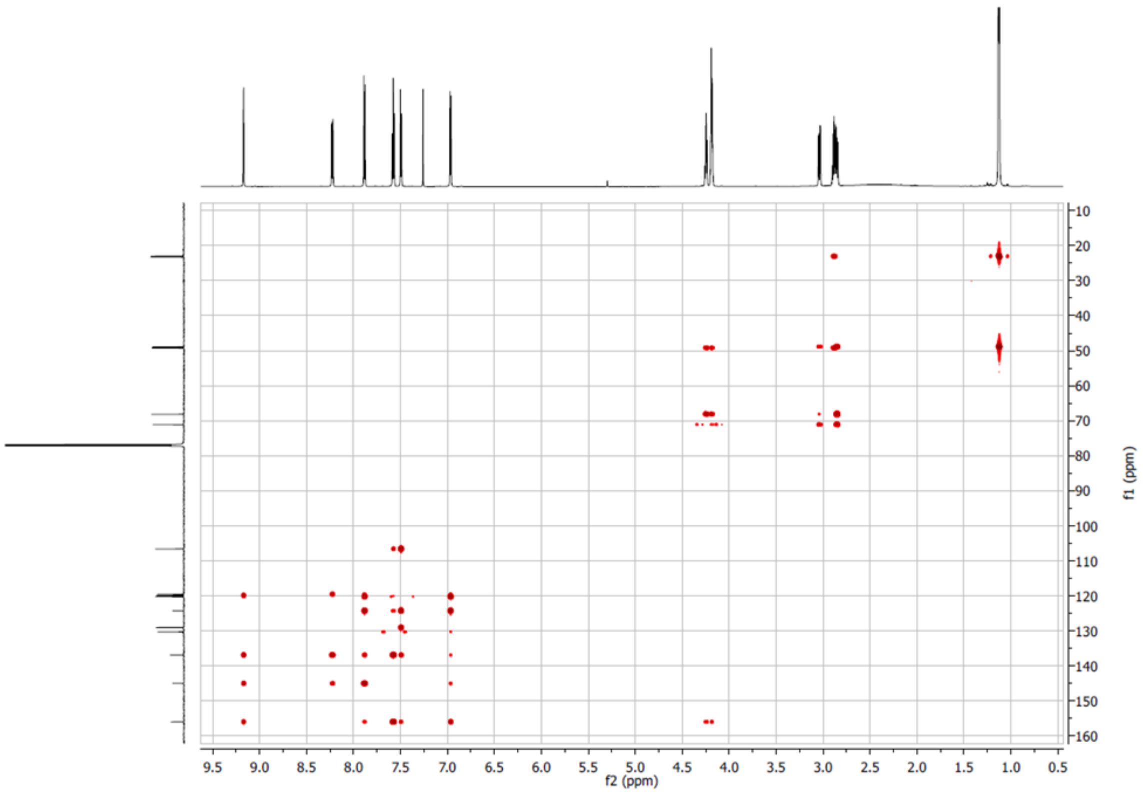

Figure S14 - HMBC 7-Nitropropranolol

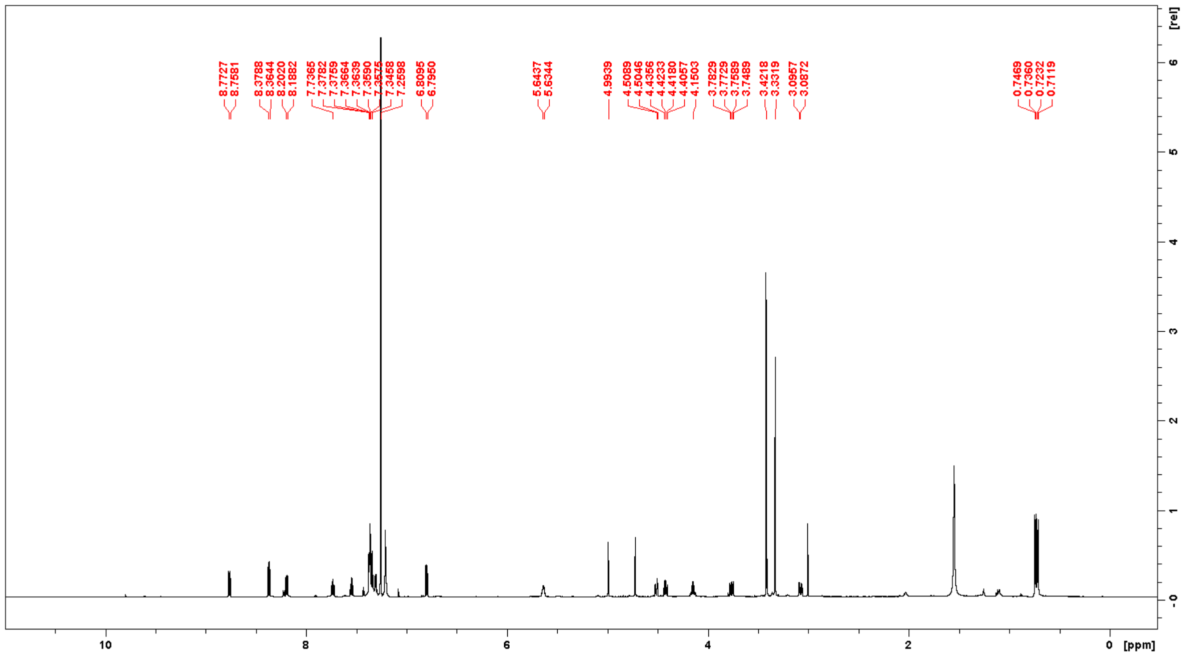

Figure S15 - ^{1}H-NMR of bis-(R)-MPA-(+)-4-NO$_2$-propranolol (CDCl$_3$)

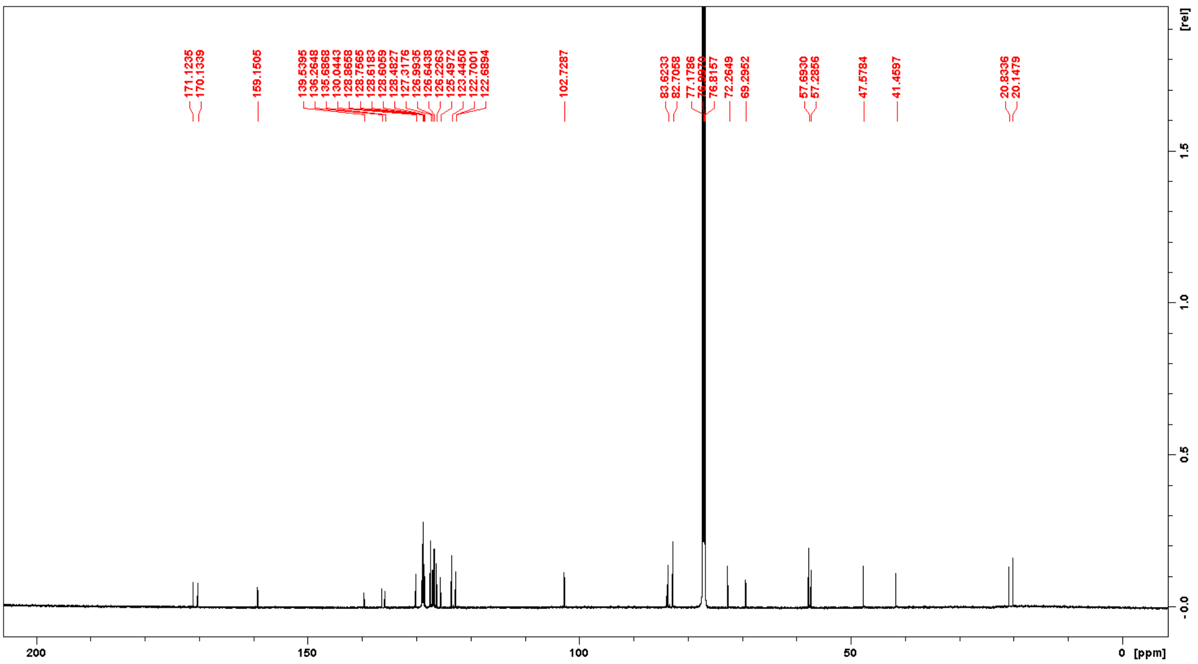

Figure S16 – ^{13}C-NMR of bis-(R)-MPA-(+)-4-NO$_2$-propranolol (CDCl$_3$)

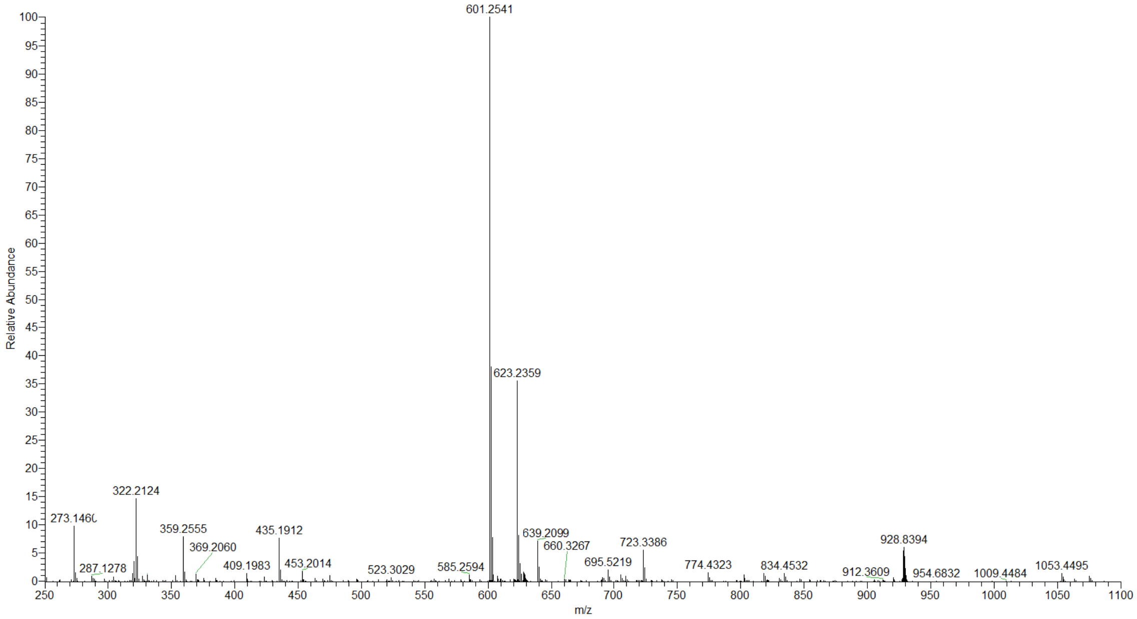

Figure S17 - HRMS spectrum of bis-(R)-MPA-(+)-4-NO_2-propranolol

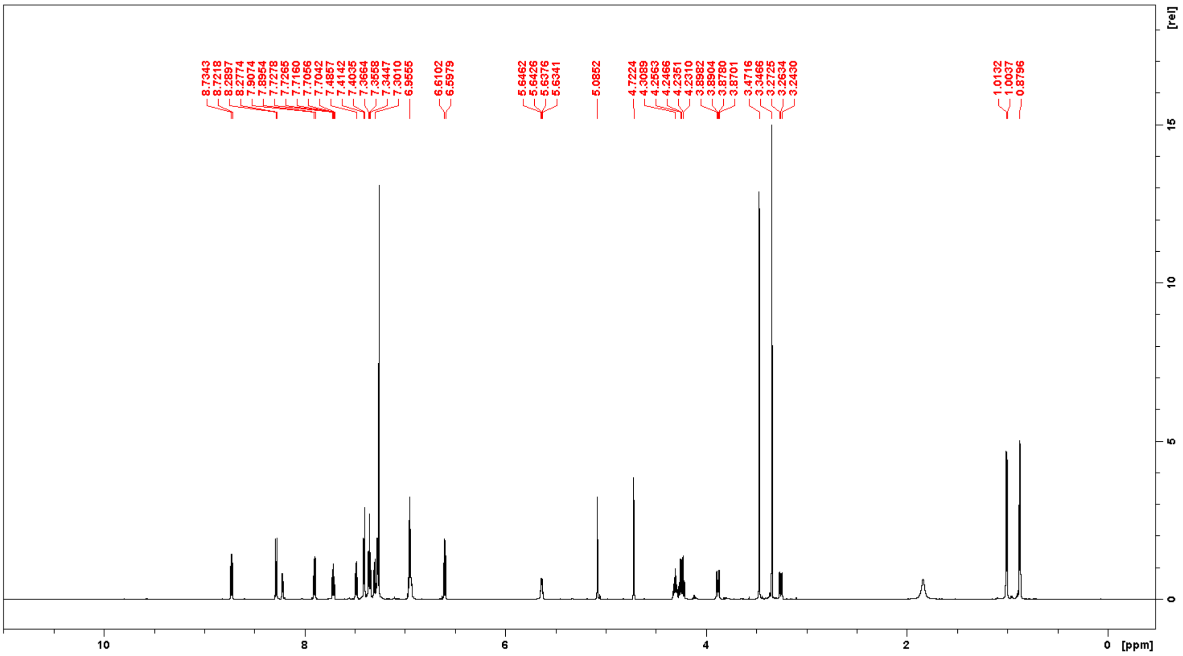

Figure S18 - ^{1}H-NMR of bis-(S)-MPA-(+)-4-NO_2-propranolol ($CDCl_3$)

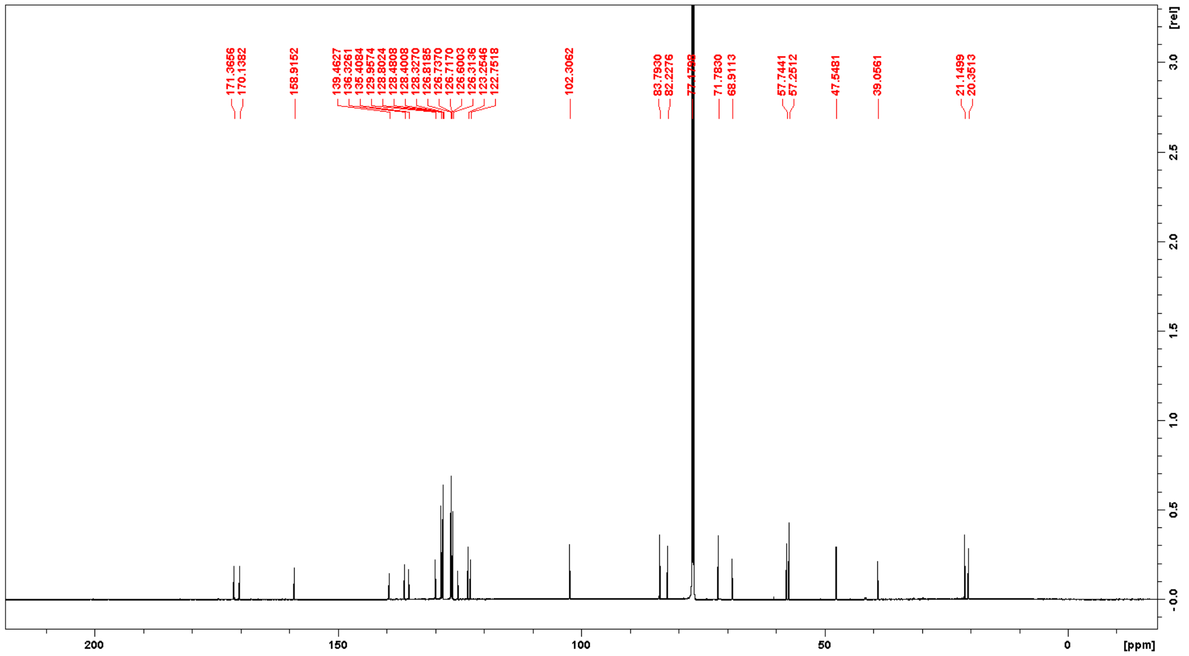

Figure S19 – ^{13}C-NMR of bis-(S)-MPA-(+)-4-NO_2-propranolol ($CDCl_3$)

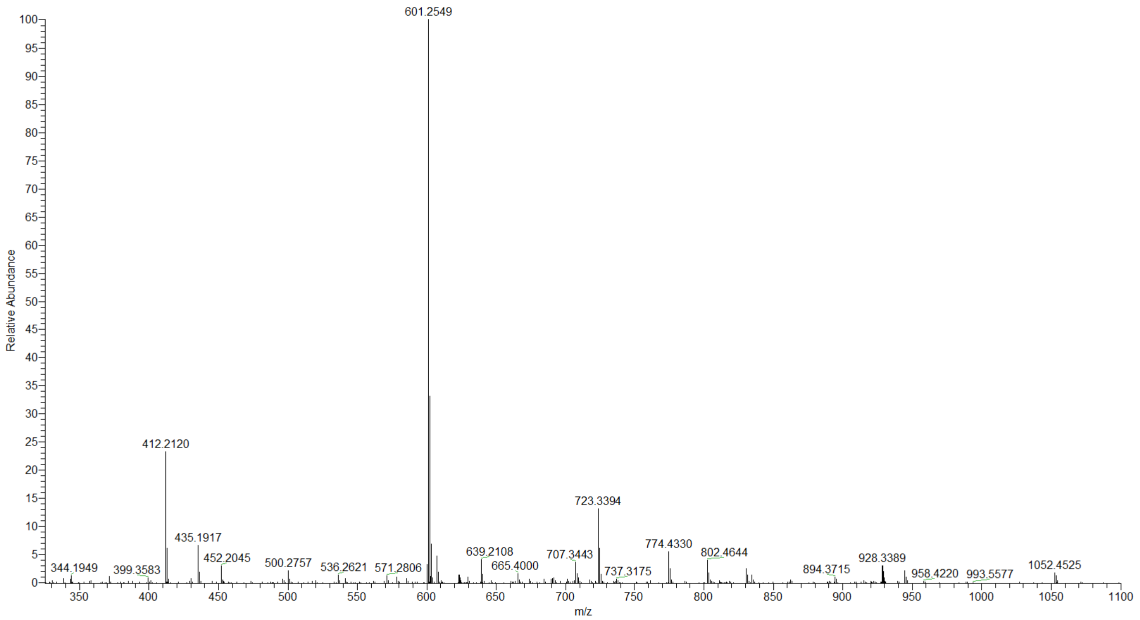

Figure S20 - HRMS spectrum of bis-(S)-MPA-(+)-4-NO_2-propranolol

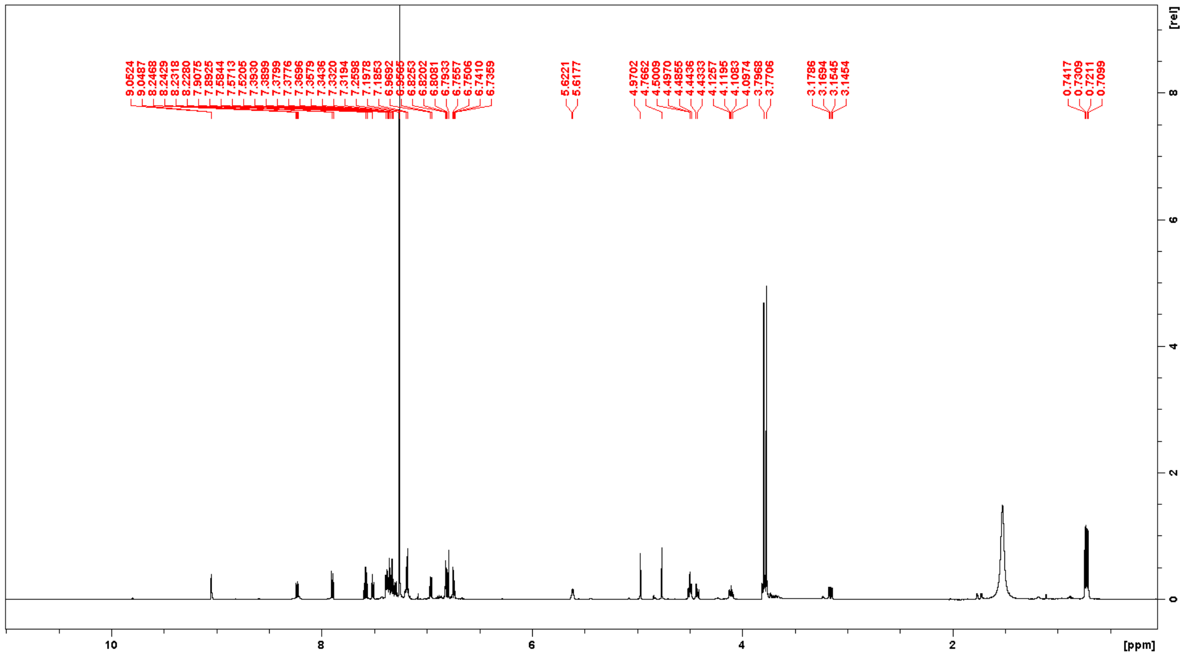

Figure S21 - ^{1}H-NMR of bis-(R)-MPA-(+)-7-NO_2-propranolol ($CDCl_3$)

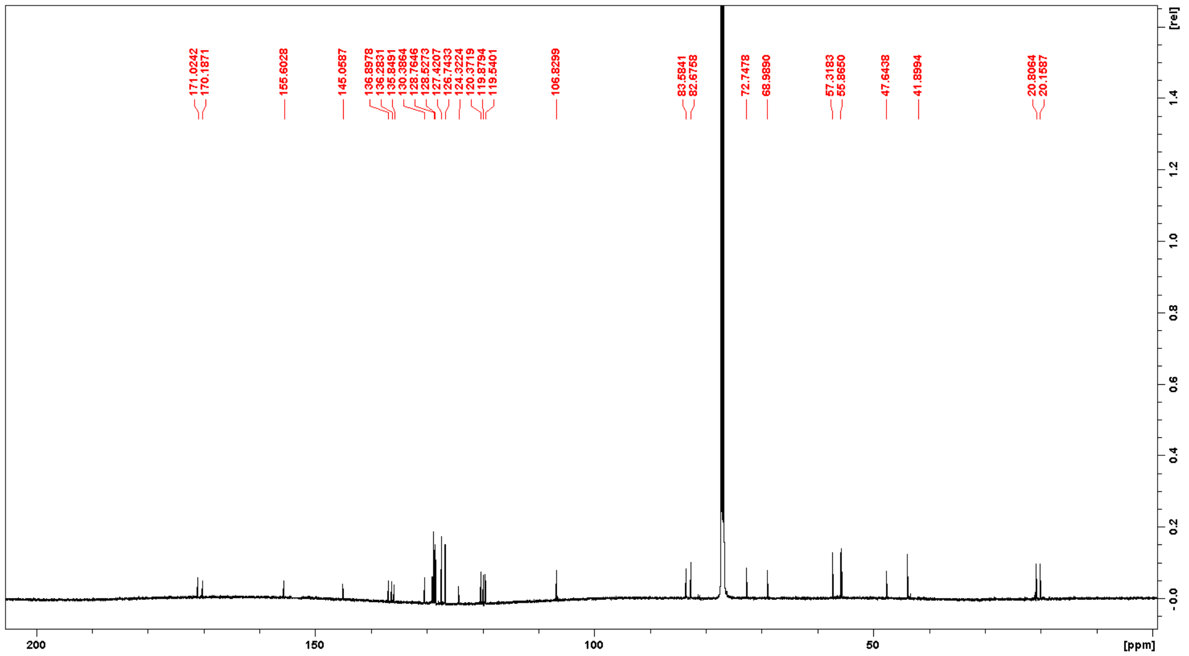

Figure S22 – ^{13}C-NMR of bis-(R)-MPA-(+)-7-NO_2-propranolol ($CDCl_3$)

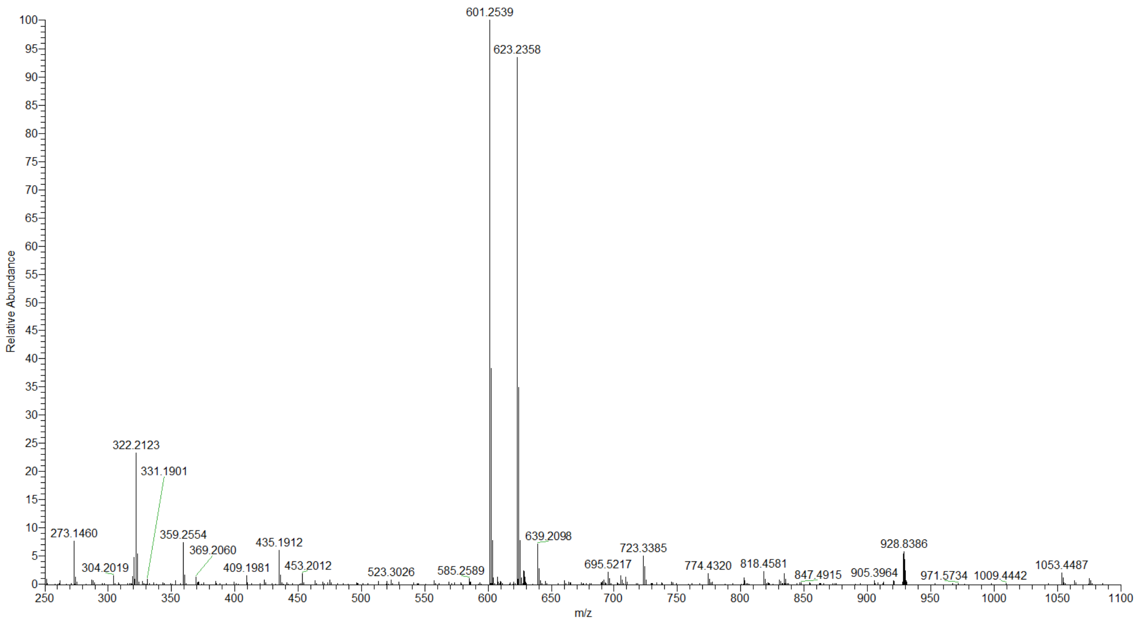

Figure S23 - HRMS spectrum of bis-(R)-MPA-(+)-7-NO_2-propranolol

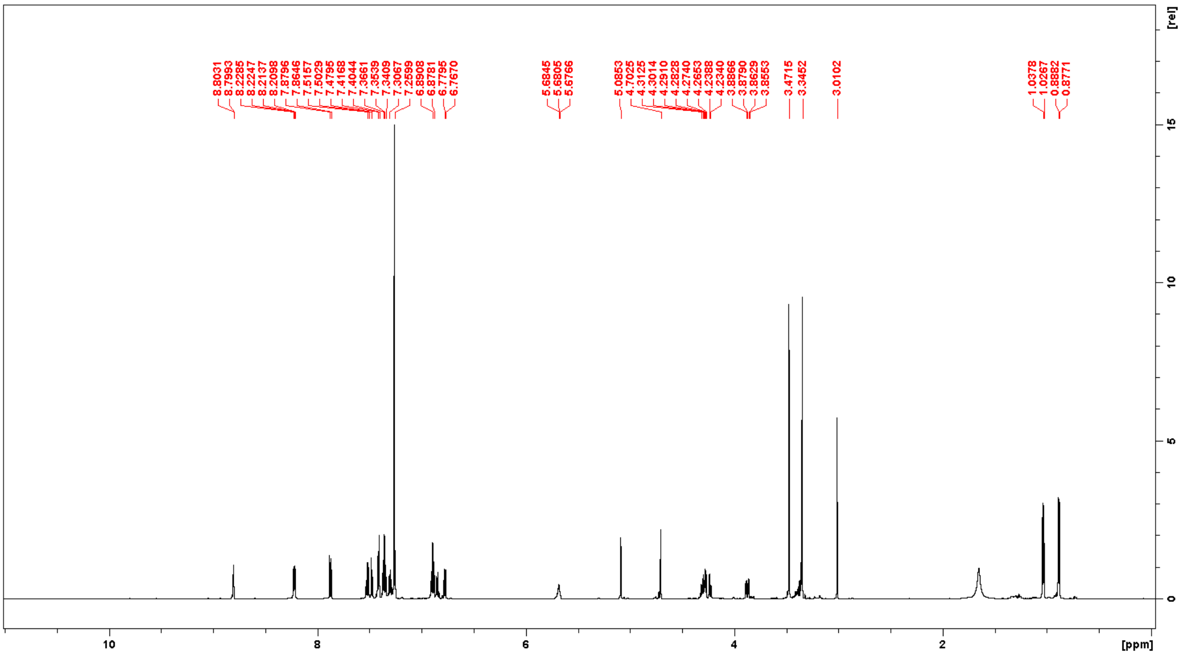

Figure S24 - ^{1}H-NMR of bis-(S)-MPA-(+)-7-NO_2-propranolol ($CDCl_3$)

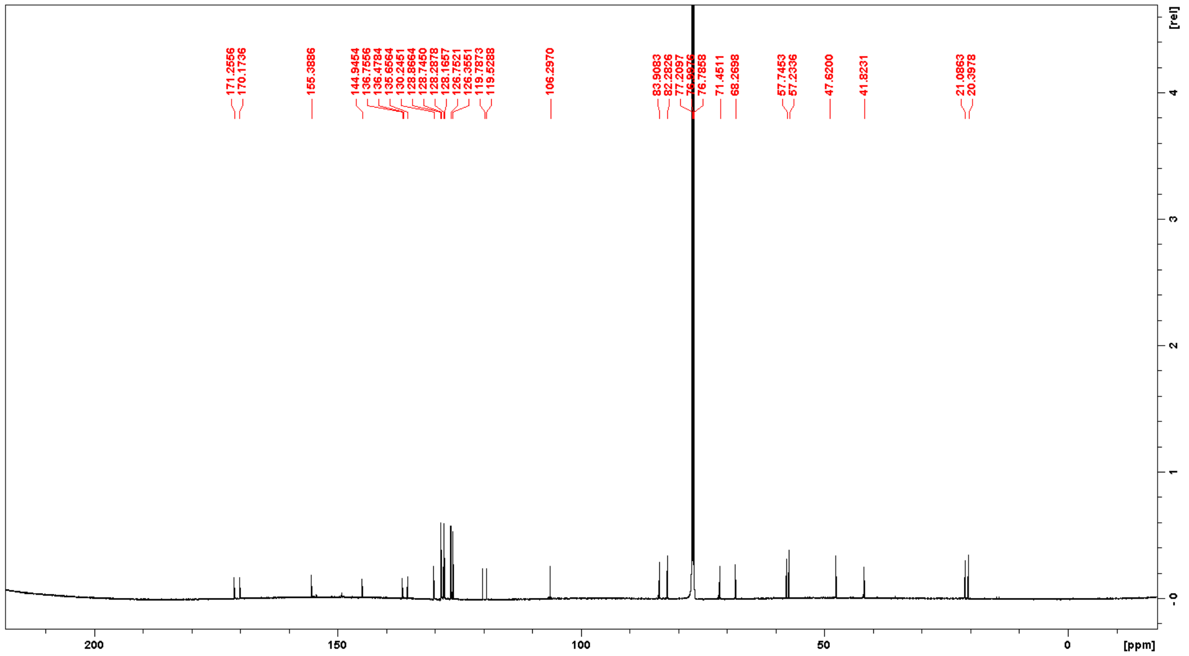

Figure S25 – ^{13}C-NMR of bis-(S)-MPA-(+)-7-NO_2-propranolol ($CDCl_3$)

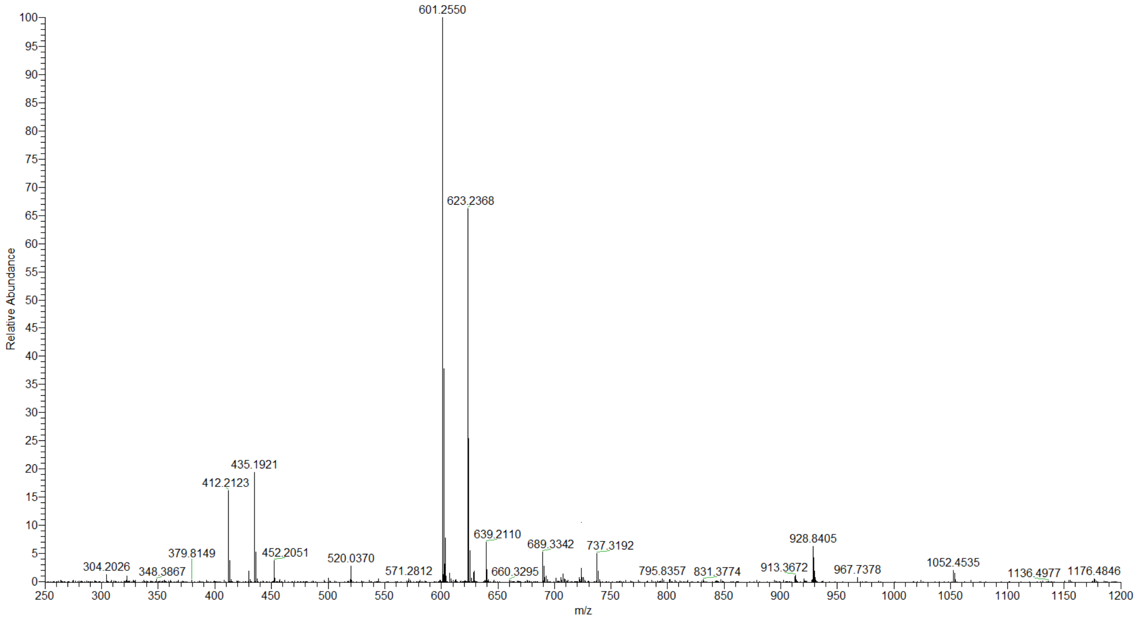

Figure S26 - HRMS spectrum of bis-(S)-MPA-(+)-7-NO_2-propranolol

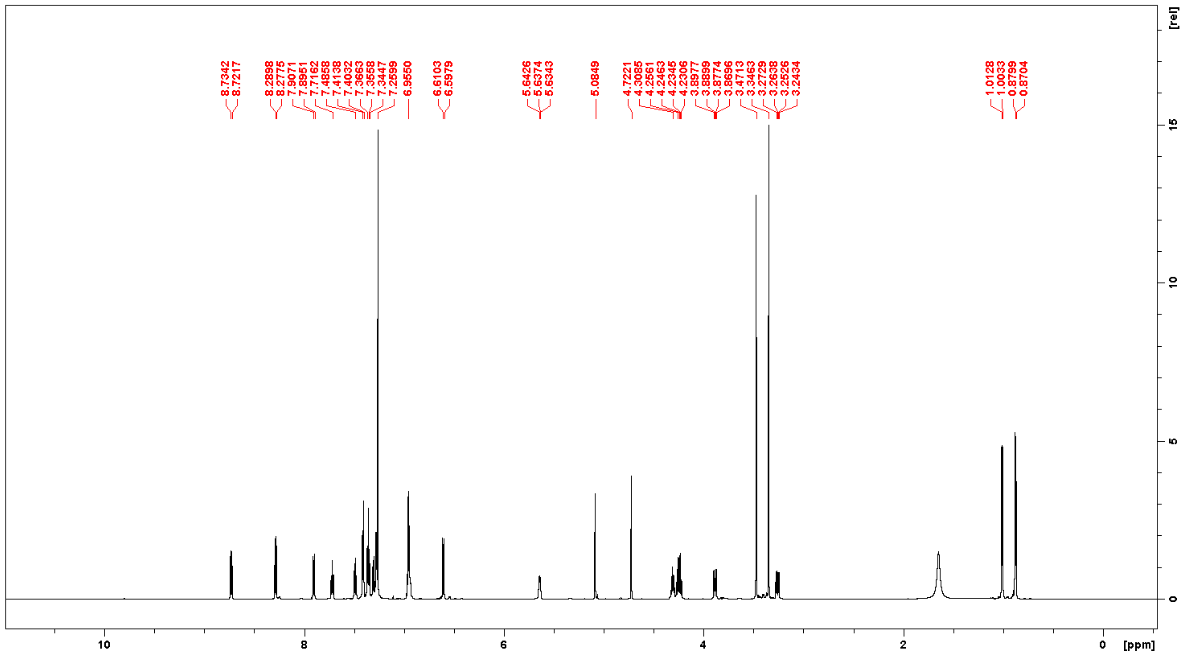

Figure S27 - ^{1}H-NMR of bis-(R)-MPA-(-)-4-NO_2-propranolol ($CDCl_3$)

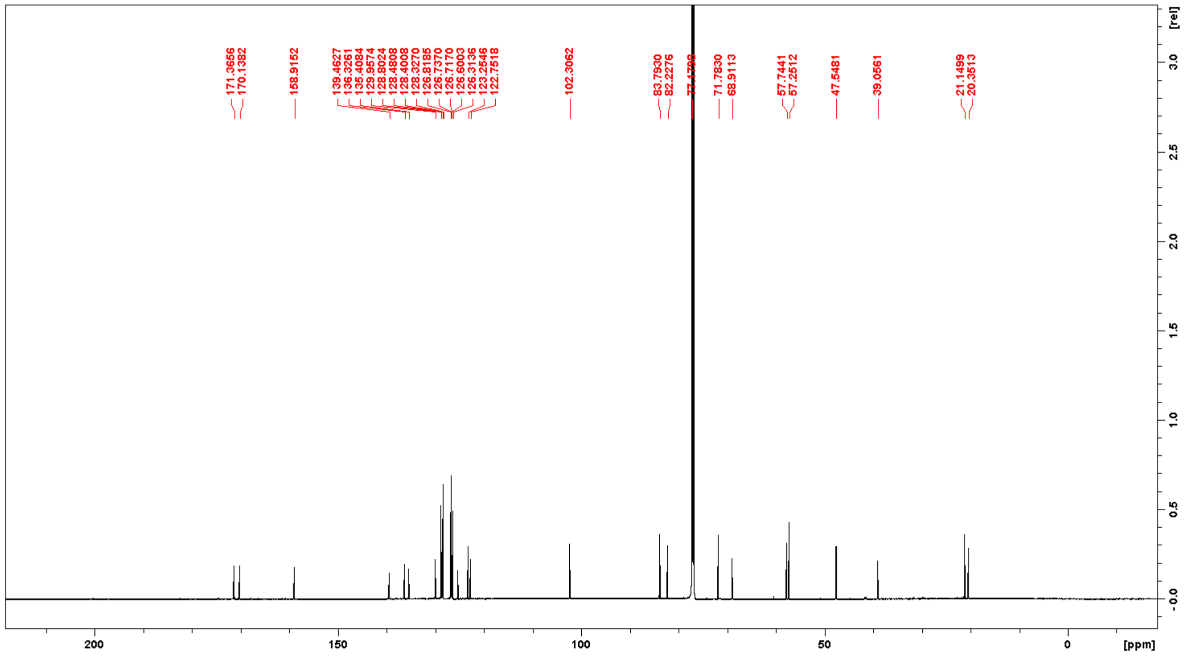

Figure S28 – ^{13}C-NMR of bis-(R)-MPA-(-)-4-NO_2-propranolol ($CDCl_3$)

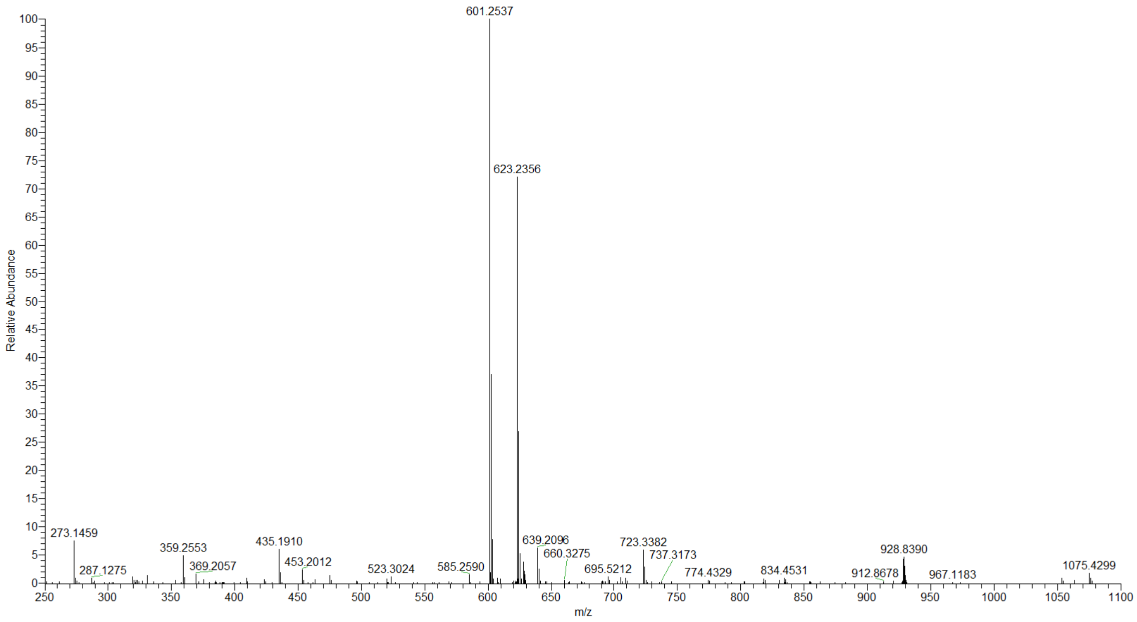

Figure S29 - HRMS spectrum of bis-(R)-MPA-(-)-4-NO_2-propranolol

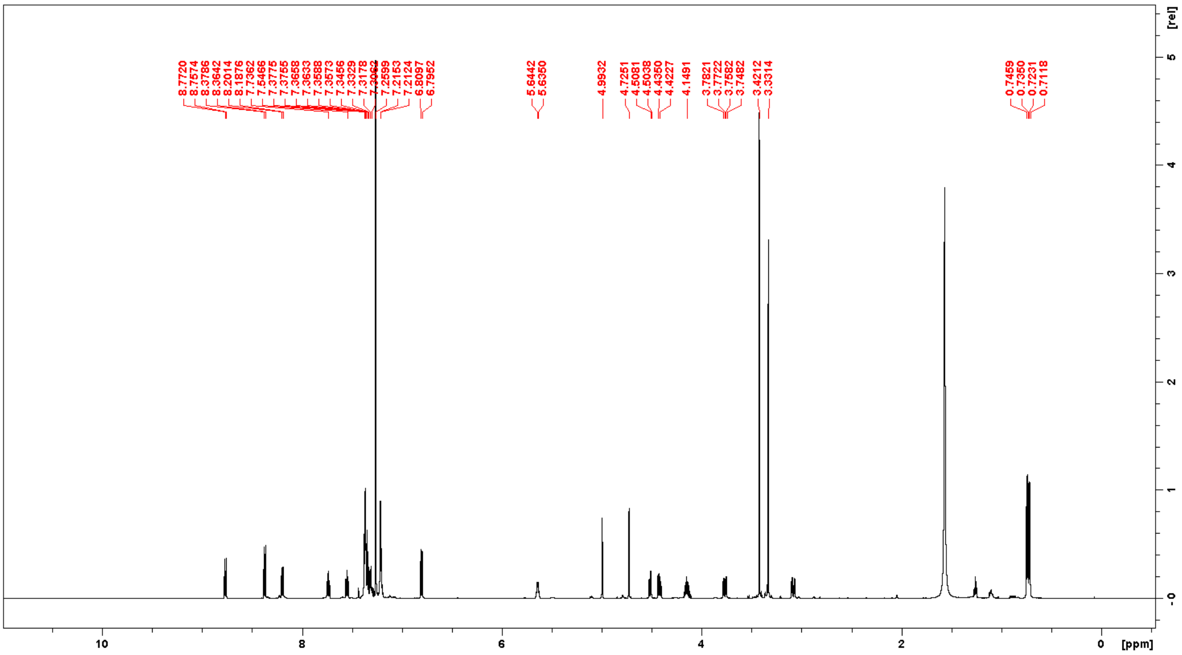

Figure S30 - ^{1}H-NMR of bis-(S)-MPA-(-)-4-NO_2-propranolol ($CDCl_3$)

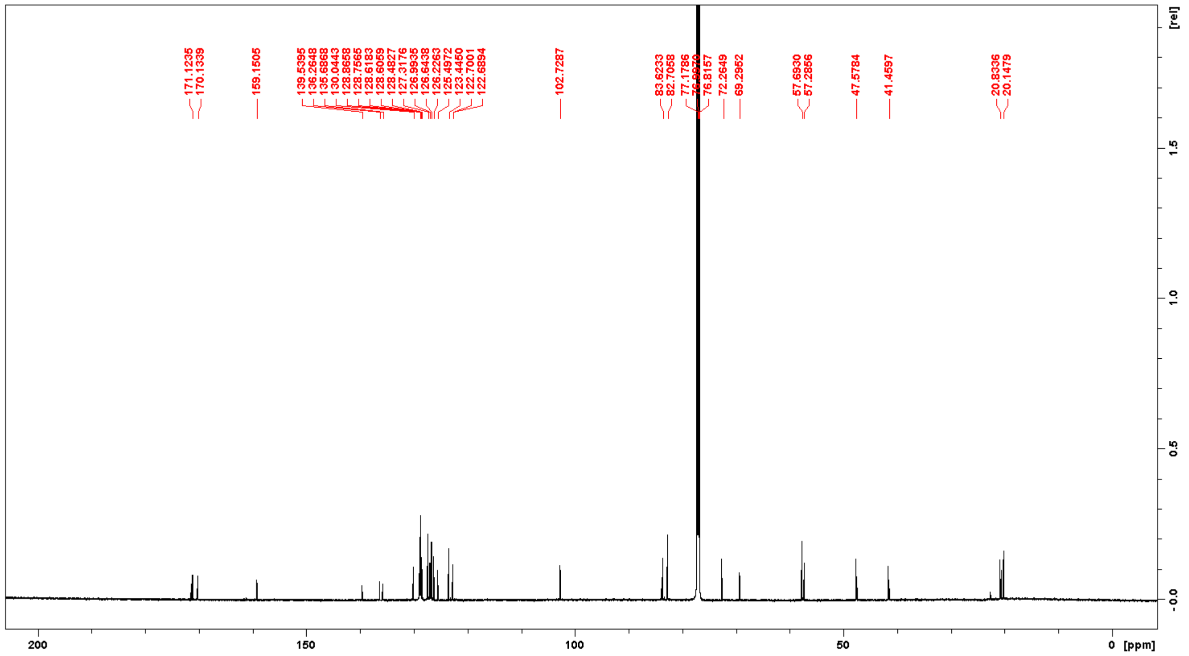

Figure S31 – ^{13}C-NMR of bis-(S)-MPA-(-)-4-NO_2-propranolol ($CDCl_3$)

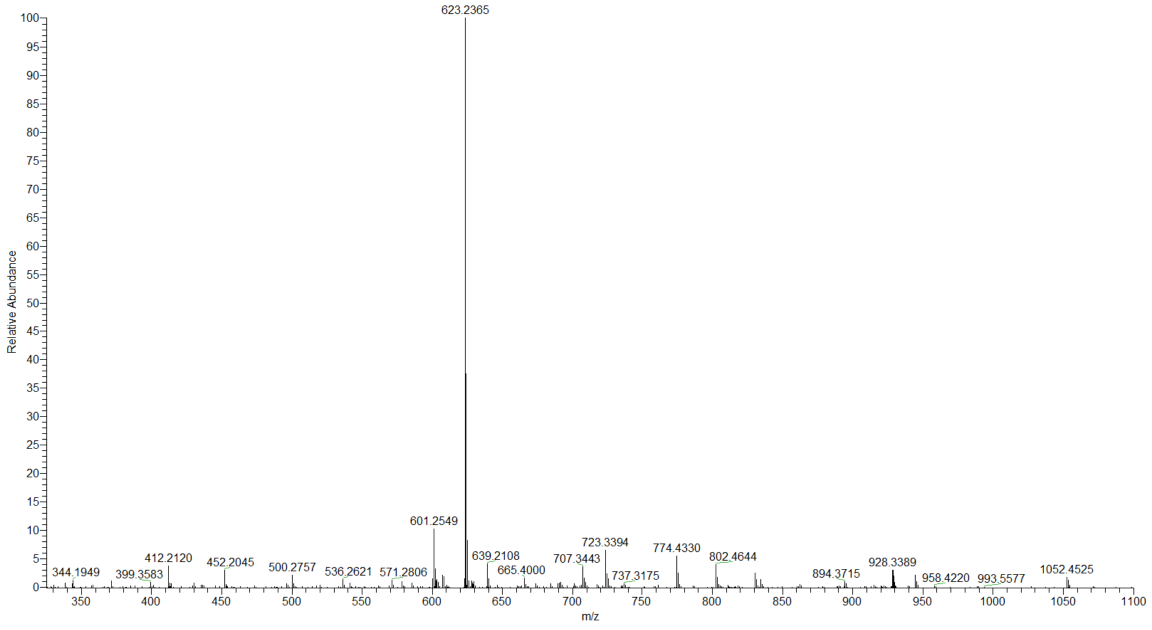

Figure S32 - HRMS spectrum of bis-(S)-MPA-(-)-4-NO_2-propranolol

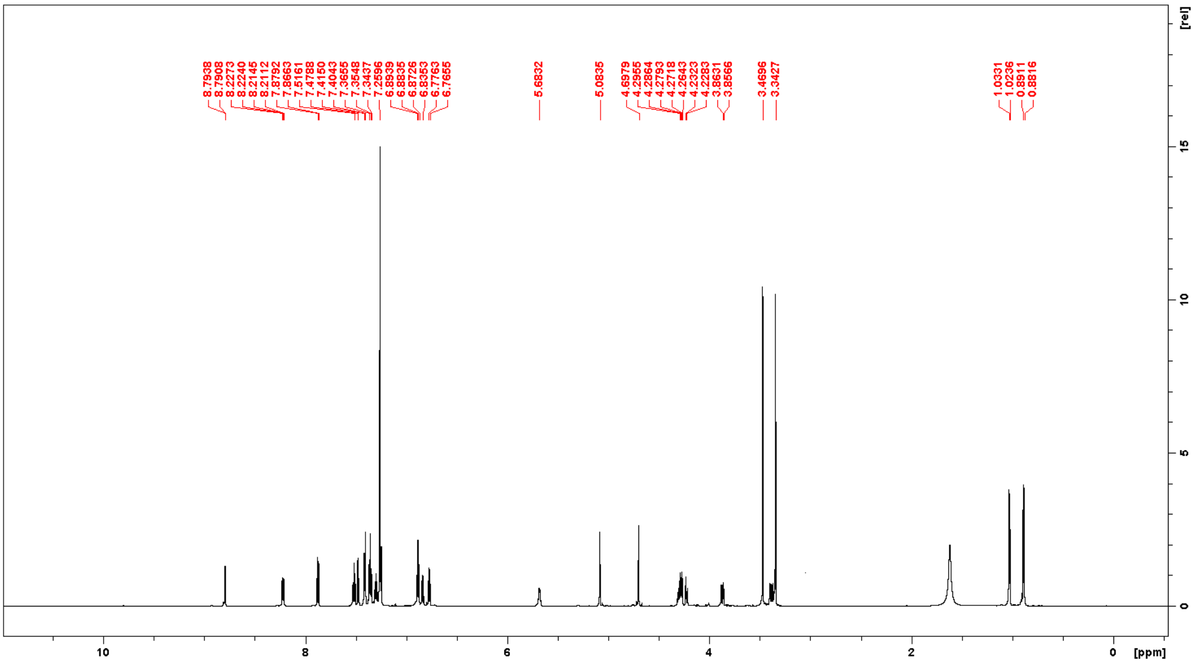

Figure S33 - ^{1}H-NMR of bis-(R)-MPA-(-)-7-NO_2-propranolol ($CDCl_3$)

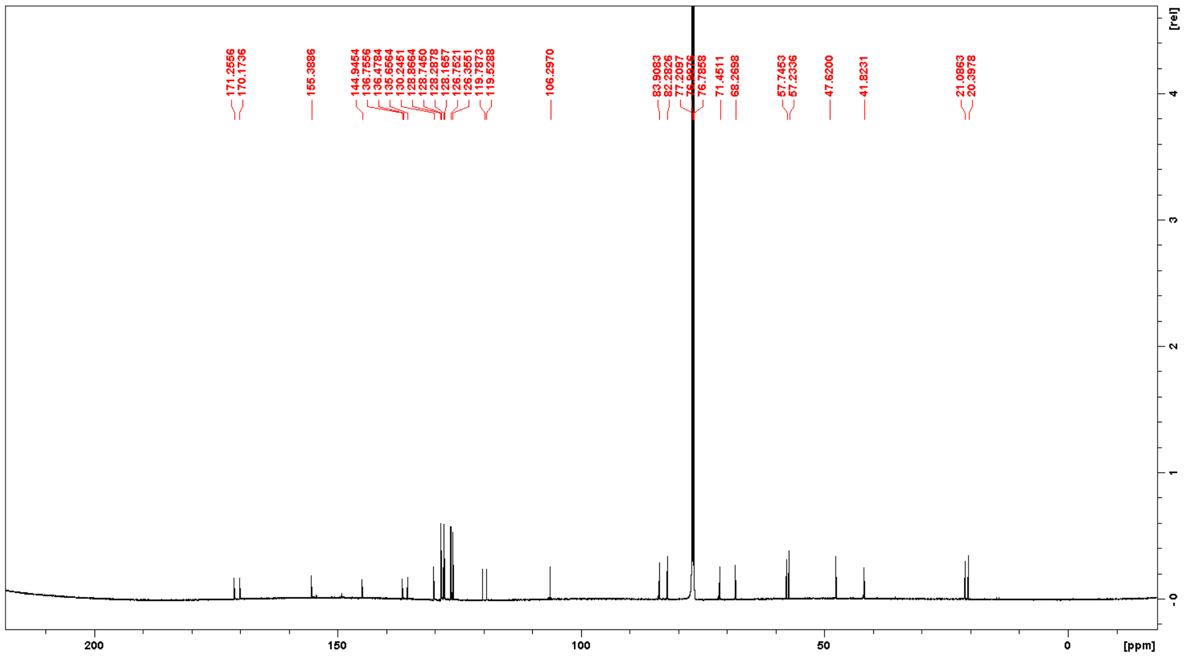

Figure S34 – ^{13}C-NMR of bis-(R)-MPA-(-)-7-NO_2-propranolol ($CDCl_3$)

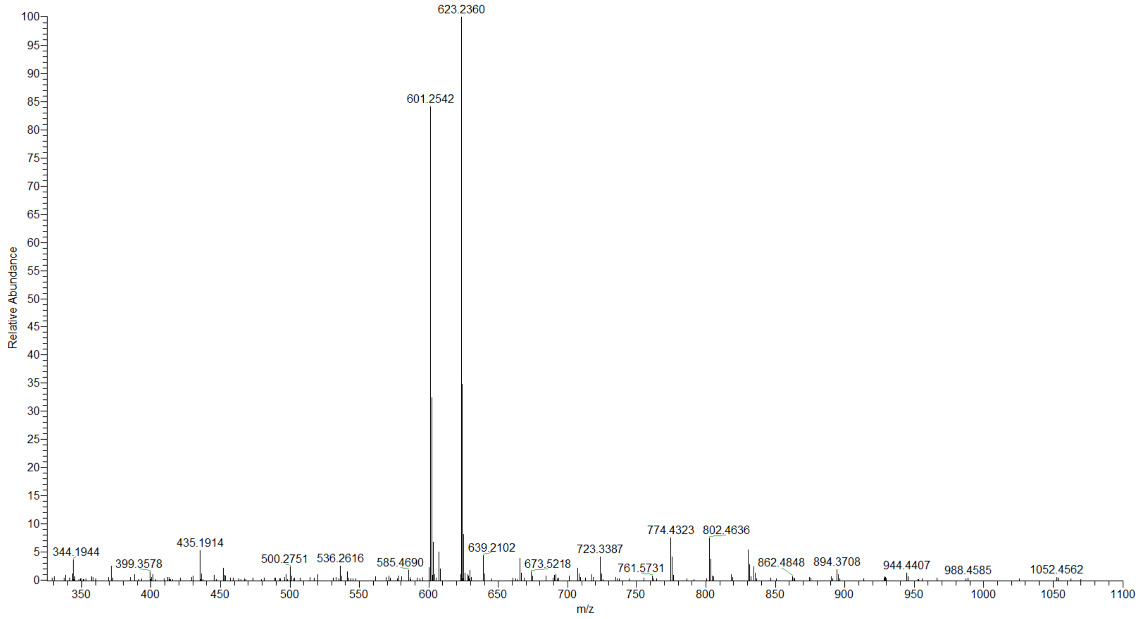

Figure S35 - HRMS spectrum of bis-(R)-MPA-(-)-7-NO_2-propranolol

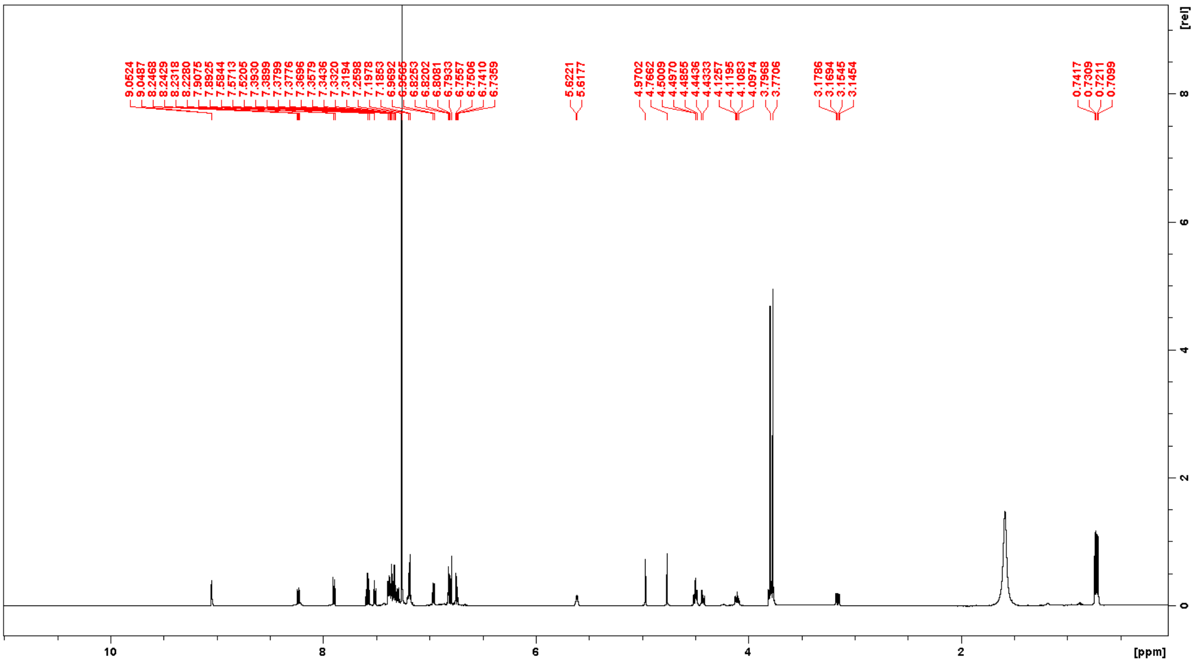

Figure S36 - ^{1}H-NMR of bis-(S)-MPA-(-)-7-NO_2-propranolol ($CDCl_3$)

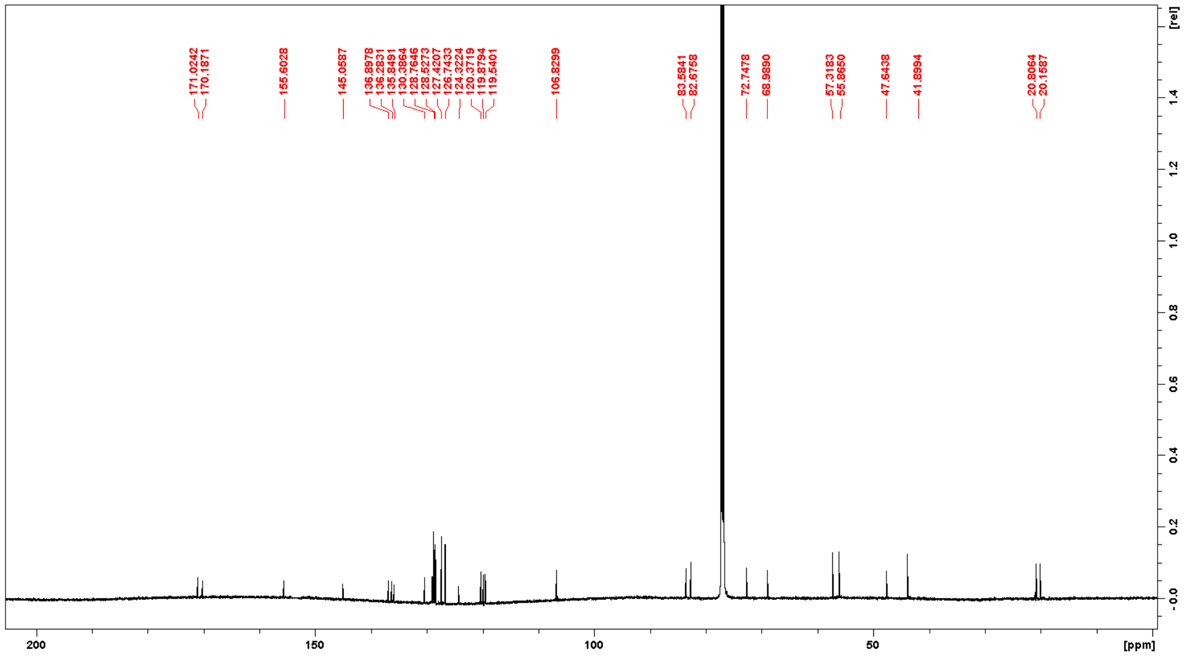

Figure S37 – ^{13}C-NMR of bis-(S)-MPA-(-)-7-NO_2-propranolol ($CDCl_3$)

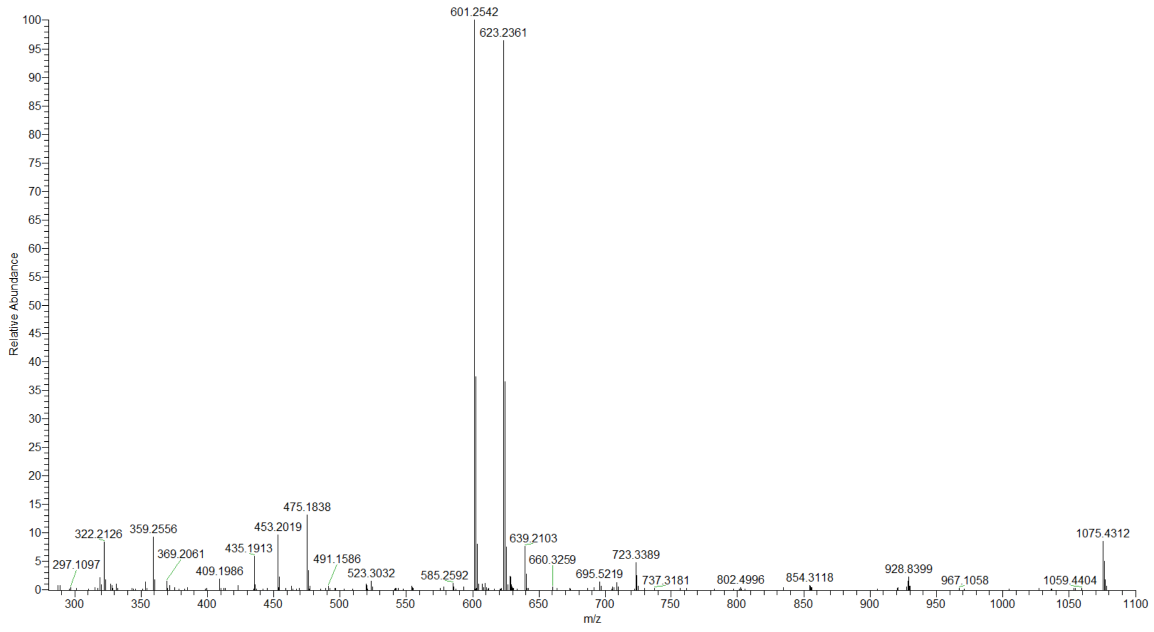

Figure S38 - HRMS spectrum of bis-(S)-MPA-(-)-7-NO_2-propranolol
